# Supplementary material for: Identification and functional analysis of non-coding regulatory small RNA FenSr3 in Bacillus amyloliquefaciens LPB-18
Source: PeerJ. 2023 May 15;11:e15236. doi: 10.7717/peerj.15236 (PMC10194069; doi:10.7717/peerj.15236)
Supplement: Supplemental Information 4 [file peerj-11-15236-s004.zip › KO/CK-vs-T1_map/map04928.html]

KEGG PATHWAY: Parathyroid hormone synthesis, secretion and action - Reference pathway


|  |  |
| --- | --- |
| **Parathyroid hormone synthesis, secretion and action - Reference pathway** |  |

[
Pathway menu
| Organism menu
| Pathway entry
| Show description
| User data mapping
]

Parathyroid hormone (PTH) is a key regulator of calcium and phosphorus homeostasis. The principal regulators of PTH secretion are extracellular ionized calcium (Ca2+) and 1,25-dihydroxyvitamin D (1,25(OH)2D3). Under conditions of dietary Ca restriction, a decrement in serum Ca concentration induces release of PTH from the parathyroid gland. PTH acts on bone and kidney to stimulate bone turnover, increase the circulating levels of 1,25(OH)2D3 and calcium and inhibit the reabsorption of phosphate from the glomerular filtrate. This hormone exerts its actions via binding to the PTH/PTH-related peptide receptor (PTH1R). PTH1R primarily activates two sub-types of heterotrimeric Gproteins: Gs and Gq , which in turn regulate the activity of adenylyl cyclases and phospholipase C (PLC) that control the flow of cAMP/PKA and IP/PKC signaling cascades, respectively.

|  |  |  |
| --- | --- | --- |
| Reference pathway | 184% 150% 122% 100% 82% 67% 55% | 图片下载 |
